# Self-rated health of university students in Germany–The importance of material, psychosocial, and behavioral factors and the parental socio-economic status

**DOI:** 10.3389/fpubh.2023.1075142

**Published:** 2023-02-10

**Authors:** Christian Deindl, Katharina Diehl, Jacob Spallek, Matthias Richter, Wiebke Schüttig, Petra Rattay, Nico Dragano, Claudia R. Pischke

**Affiliations:** ^1^Department of Social Sciences, TU Dortmund University, Dortmund, Germany; ^2^Department of Medical Informatics, Biometry and Epidemiology, Chair of Epidemiology and Public Health, Friedrich-Alexander-Universität Erlangen-Nürnberg (FAU), Erlangen, Germany; ^3^Department of Public Health, Brandenburg University of Technology Cottbus-Senftenberg, Senftenberg, Germany; ^4^Department of Sport and Health Sciences, Technical University of Munich, Munich, Germany; ^5^Department of Epidemiology and Health Monitoring, Robert Koch Institute, Berlin, Germany; ^6^Institute of Medical Sociology, Centre for Health and Society, University Hospital and Medical Faculty, Heinrich-Heine-University Düsseldorf, Düsseldorf, Germany

**Keywords:** students, health, university, inequality, National Educational Panel Study

## Abstract

**Introduction:**

Health inequalities start early in life. The time of young adulthood, between late teens and early twenties, is especially interesting in this regard. This time of emerging adulthood, the transition from being a child to becoming an adult, is characterized by the detachment from parents and establishing of an own independent life. From a health inequality perspective, the question about the importance of the socio-economic background of parents is important. University students are an especially interesting group. Many students come from a privileged background and the question of health inequality among university students has not yet been properly studied.

**Methods:**

Based on the National Educational Panel Study (NEPS), we analyzed health inequalities among 9,000 students in Germany (∅ 20 years in the first year of their studies) over a period of 8 years.

**Results:**

We found that most university students (92%) in Germany reported a good and very good health. Yet, we still found substantial health inequalities. Students whose parents had a higher occupational status reported less health problems. Additionally, we observed that health inequalities had indirect impact on health via health behavior, psychosocial resources, and material conditions.

**Discussion:**

We believe our study is an important contribution to the understudied subject of students' health. We see the impact of social inequality on health among such a privileged group like university students as an important sign of the importance of health inequality.

## 1. Introduction

The period between late teens and early twenties, often referred to as emerging adulthood (1), is a critical phase in life where important developmental steps are taken (2), including the completion of education and the transition from school to work. This is especially true for university students who are in the final chapter of their educational journey and close to entering the labor market. In addition to their educational and occupational development, they are also in the process of establishing their own independent life away from the parental home (3). They have to adapt to a new and often challenging university environment during the beginning of their studies.

Overall, the physical health of university students, as most being in their late teens or early twenties, is reasonably good but a little bit lower than of their non-student peers (4). Nonetheless, there is some evidence for consistent health problems and indication of a correlation between health conditions and socio-economic status of students. In Germany, Diehl et al. (5, 6) found that subjective social status was associated with different health indicators, i.e., those with a lower status displaying greater health problems overall compared to those with a higher status. In a large health survey including 6,198 German university students, Grützmacher et al. also found that although students are overall quite healthy, they rated their health lower and seemed to consume more drugs and alcohol in comparison with their non-student peers (4, 7). Eisenberg et al. (8) found evidence for mental health problems among university students. Sixteen percent of undergraduate and 13 % of graduate students showed symptoms of depression or anxiety. They also noted that these two psychological disorders were more prevalent among students with a lower socio-economic status. Ibrahim et al. (9) found in a systematic review that the rates of depression of university students were indeed higher than in the general population. For students in the UK, the risk of depression was higher for those from a more disadvantaged background (10). Steptoe et al. (11) found similar results in a comparison of 23 countries. They also found that income inequality and the level of individualistic culture had an impact on depression among university students (11).

Social determinants are important factors for health differences (12) and are of course also found for university students (6). As university students have not yet developed a stable socio-economic status of their own, the socio-economic background of their parents is a major cause for health inequalities in young adults (13). The impact of the socio-economic position on health can therefore be best described as the impact of the highest socio-economic position of parents on health of their offspring. Additionally, Hagquist (14) proposes to distinguish between the socio-economic position students try to achieve and not to concentrate on the socio-economic position of their parents.

Most of these results show the importance of social inequality for students' health. The main pathways of social inequalities in health are behavioral, psychosocial and material factors (15–17). The harmful outcome of smoking, drinking and overweight is prominent in the literature (3) and well documented for university students (18). Research shows that these behavioral aspects can vary by socio-economic status (3, 19–22). Psychosocial aspects can be responsible for causing health problems (23) which are socially patterned as well (24). The harmful effects of psychosocial conditions, such as psychosocial stress on health, have been previously described in the effort-reward model of work stress (25) and were also found for university students (26). Thirdly, less advantageous material conditions are likely to cause unhealthy living conditions, such as an increased exposure to traffic, loud noise or inadequate housing (27).

Whether the socio-economic background of parents still has a significant impact on students' health independent or in combination with behavioral, psychosocial and material factors in German university students is still unclear. Our study therefore had three main objectives. First, we described students' health over the course of the time of their studies and analyzed the impact of parents' socio-economic status on self-rated health. Second, we analyzed the impact of behavioral, psychosocial and material factors on students' self-rated health, and third, we analyzed the possible impact of parents' socio-economic status in students' self-rated health *via* health behavior, psychosocial, and material resources.

## 2. Data and method

We used data from the 2020 data release (15.0.0, May 2020) of the National Educational Panel Study [NEPS; see (28, 29)]. The NEPS is carried out by the Leibniz Institute for Educational Trajectories (LIfBi, Germany) in cooperation with a nationwide network. The NEPS study is conducted under the supervision of the German Federal Commissioner for Data Protection and Freedom of Information (BfDI) and in coordination with the German Standing Conference of the Ministers of Education and Cultural Affairs (KMK) and–in the case of surveys at schools–the Educational Ministries of the respective Federal States. All data collection procedures, instruments and documents were checked by the data protection unit of the Leibniz Institute for Educational Trajectories (LIfBi). The necessary steps were taken to protect participants' confidentiality according to national and international regulations of data security. Participation in the NEPS study was voluntary and based on the informed consent of participants. This consent to participate in the NEPS study can be revoked at any time.

NEPS provided data for six cohorts across the life course: newborns, children in kindergarten, children in the 5^th^ and 9^th^ grade, students at universities and adults in Germany. Our analyses were based on the starting cohort 5 consisting of university students. The data collection for this cohort started in the winter term of the academic year 2010/11 with a representative sample of 17,909 first-year university students. For most years, NEPS conducted two interviews per year, one computer-assisted telephone interview (CATI) and one computer-assisted web-interview (CAWI). The CATI-interview usually took place in the spring or summer and the CAWI in the autumn of the same year, with the exception of 2017, where both types of interviews were conducted simultaneously. Currently, there were 15 waves of data available: nine CATI waves and six CAWI waves. Starting cohort 5 started with 17,909 respondents in 2010/11. Due to panel mortality, the number of respondents was reduced to 6,531 after 15 waves (nine years later). The age structure was quite heterogeneous, with students being between the ages of 16 and 64 years with a mean age of 21 years in the first year of the survey. Since our focus was on young adults' health, we excluded all students who were older than 25 years in the first wave from the analysis. Furthermore, students who finished their studies were kept in the analysis as long as they did not drop out of NEPS. This left us with 24,394 observations of 8,898 students over a maximum period of 9 (CATI) waves for our main analysis.

As we described in the following sections, some variables in NEPS were assessed irregularly. Some were asked only once, some were asked with long intervals between them, some were asked only in later waves of NEPS. Therefore, we divided our analysis in two parts: a cross-sectional and a longitudinal part. Our cross-sectional analysis captured conditions at the beginning of the studies; the longitudinal analysis captured the development of students' health over time. To maximize our samples sizes for each analysis, we used the full available sample for each analysis resulting in slightly different sample sizes for each model. See Table 1 for an overview of the availability of key-independent variables in NEPS.

**Table 1:** Availability of key independent variables and original sample size for each wave.

**Year**	**Type**	**N**	**BMI**	**Smoking**	**Drinking**	**Success**	**Support**	**Self-esteem**	**Cost of studies**
2010/11	CATI	17,908				X			X
2011	CAWI	12,248					X		
2012	CATI	13,113	X	X	X			X	
2012	CAWI	11,202							
2013	CATI	12,693				X			X
2013	CAWI	10,182					X		
2014	CATI	9,610		X	X				
2014	CAWI	8,628					X		
2015	CATI	10,096							
2016	CATI	9,089	X	X	X			X	
2016	CAWI	7,020					X		
2017	CATI	8,550							
2018	CATI	7,293	X						
2018	CAWI	5,161					X		
2019	CATI	6,531							

X, variable available for this wave.

### 2.1. Dependent variable

Our dependent variable was self-rated health. Respondents were asked to rate their health as very good, good, moderate, poor, or very poor. Self-rated health is a suitable measure for individual health that captures a variety of health problems [see (30) or (31) for detail on self-assessed health] and is an overall good indicator for “true” health (32). We treated self-rated health as a quasi-metric variable with higher values indicating better health. As basic assumptions of regression models for ordinal variables, especially the assumption of proportional odds, are often violated [see (33) for details on ordinal regression models] and linear regression models can be seen as “a sensible default” (34). Given our considerable large sample size possible problems associated with using linear regression models for ordinal variables (e.g., non-normally distributed outcomes) are not evident (34). Additionally, our analytical approach required a comparison of coefficients across models, something that is notoriously difficult to do on the basis of (ordinal) logistic regression models (35, 36). Self-rated health was available for all main waves in NEPS.

### 2.2. Independent variables

For the socio-demographic background of students, we used information about their age (metric), gender (female, male) and their migration background. We distinguished between students without any experience of migration, students who migrated themselves (first generation), and students who had parents (second generation) or grandparents (third generation) who migrated to Germany.

We furthermore distinguished between students studying at a University or a University of Applied Sciences and between the various study subjects: Linguistics or Cultural Studies, Law, Economics, Social Sciences, Mathematics or Sciences, Medicine, Agricultural-, Forest- and Nutrition Sciences or Veterinary Medicine, Engineering, and Arts or Fine Arts. This information was taken from the first wave of NEPS.

### 2.3. Socio-economic status

The socio-economic status was measured using occupation and education of parents. Occupational status of parents was a simple measure of the International Socio-Economic Index of Occupational Status (ISEI) that was calculated by the NEPS team. The ISEI is derived from the International Standard Classification of Occupations (ISCO 08) and translates to a metric scale ranging from 16 up to 90, where a higher value represents a higher occupational status [see (37) for detailed information about ISEI]. Our measure of education of parents distinguished between primary, lower-secondary, upper-secondary, and tertiary education. For both education and occupation, we chose the highest occupational and educational status of either father or mother. All information about parents was collected in the first wave and was transferred to all following waves.

In the first and fifth wave, students were asked “How difficult is it for you and your family to pay for the things you need for your degree course, for instance, travel costs, books or tuition fees?”. The possibilities were very difficult, rather difficult, neither nor, rather easy or very easy. Due to a low number of students describing financing their studies as very or rather difficult, these two answers were combined. Information from wave 1 was transferred to wave 2 until wave 4 and the information from wave 5 was transferred to all following waves from wave 6 till wave 15. A measure of income was unfortunately not available. Only for later waves, once students entered the labor market, a measure of income became available. This could therefore not be used.

### 2.4. Health relevant behavior

Health relevant behavior was captured by analyzing smoking behavior, alcohol consumption and the Body-Mass-Index (BMI) as an indicator for (problematic) eating behavior. Smoking was asked in waves 3, 7, and 10 using the following item: “Did you smoke in the past or do you currently smoke?” with the possible answers: have never smoked, did smoke before, currently smoke occasionally, currently smoke every day. Alcohol consumption was also asked in waves 3, 7, and 10. The question was “How often do you consume alcoholic drinks?” with the possible answers: (almost) never, once a month or less, twice or three times a month, once a week, several times a week, (almost) every day. BMI was calculated by using the information about the height and the weight of survey participants. This information was available for waves 3 and 10. Missing information was transferred from waves 3, 7 and 10 to all other waves. Information from wave 3 was transferred to wave 1 and 2 and to wave 4 until 6, information from wave 7 was transferred to waves 8 and 9, and information from wave 10 was transferred to wave 11 until wave 15.

### 2.5. Psychosocial factors

The NEPS data offered some variables capturing different health-related psychosocial aspects, such as confidence, social support, and self-esteem. Confidence was captured by the question: “How likely is it for you to successfully complete a course of study?” with the given possibilities is it very unlikely, rather unlikely, ~50:50, rather likely, or very likely. The categories very unlikely to 50:50 were grouped together, as only few students rated their likelihood this low. This variable was available for waves 1 and 5 and was transferred to all subsequent waves in the same way as described above. Social support was measured with the question: “In general, students support each other.” with the possible answers not true at all, mostly not true, partly true, mostly true and exactly true. The three lowest categories were grouped together. This variable was available for waves 2, 6, 8, 11, and 14 and was transferred to all subsequent waves as described above. Finally, self-esteem was measured based on the question: “All in all, I am satisfied with myself.” with possible answers with a range from 13 to 50, where higher values indicated a higher level of satisfaction. This variable was available for waves 3 and 10 and transferred to all other waves as described above.

### 2.6. Analysis

We had three analytical aims: first, we wanted to describe the health of German university students and analyse the impact of parents' socio-economic status, second, we wanted to analyse the impact of behavioral, psychosocial and material factors on students' health, and third, we wanted to analyse the indirect impact of their socio-economic background *via* health-related behavior, psychosocial factors, and material conditions. Our first and second aims were achieved by performing descriptive and multivariate analyses. The multivariate analyses disentangled the impact of the socio-economic status of parents, and health-related behavior, psychosocial factors, and material conditions on students' health over time. For this reason, we applied linear multilevel models. Multilevel models are, in principal, an extension of an ordinary linear regression model. Multilevel models allow variables to vary across time (i) and person (j) and include an error term for the different level, in our case time and person-level *y*_*ij*_ = β_0*ij*_+ β_1*ij*_*x*_1*ij*_+ β_2*j*_*x*_2*j*_+*e*_*ij*_+*u*_*j*_ [see (38) for details on multilevel analysis].

To disentangle direct and indirect effects of socio-economic conditions and health, we applied a mediation approach. Unfortunately, due to the fact that the mediator variables are categorical variables with multiple categories we couldn't apply a standard mediation analysis using path models. We therefore did our mediation analysis in three steps. In a first step, we correlated our mediation variables with our independent socio-economic variables (education and occupation of parents) using multinomial regression models. For this approach, we used the information from the first wave our mediation variable appeared in the NEPS. For most variables, this was the first, the second or third wave (see above for details on the first appearance of variable in the NEPS). In our second step, we used these results to group the values of our variables according to their sensitivity to socio-economic status. If, for example, a higher socio-economic background correlated positively with daily drinking, students who drank daily were grouped into “drinking behavior of higher social class.” If there was no significant correlation with a certain value, this was coded as independent from socio-economic background. In our third and last step, these new variables were then used as independent variables for multilevel regression models on self-rated health. Through this approach, we were able to incorporate the direct and indirect impact of socio-economic conditions on health.

## 3. Results

In the first year of the NEPS-student-cohort, students in our analytic sample had a mean age of 20 years and were between 16 and 25 years old (see Table 2). Roughly, 40% of first year students in NEPS were male. Eighty Two percent had no migration background, nearly 3% were migrants themselves and 7% had parents or grandparents who had migrated to Germany. Twenty-Two percent of the students started their studies at a University of Applied Sciences. The majority of students studied Linguistics or Cultural Studies, Mathematics and Sciences were on second place, followed by Engineering, Economics, Social Sciences, Medicine, Law, Arts and Fine Arts, and Agricultural-, Forest- and Nutrition Sciences and Veterinary Medicine.

**Table 2:** Descriptive statistics for initial characteristics of students in 2010, 2011, and 2012.

	**%/$\bar{x}$**		**%/$\bar{x}$**
**Health (2010)**		**Health-related behavior**	
Very bad	0.16	**BMI (2012)**	
Bad	0.75	Underweight	5.67
Medium	7.26	Normal weight	78.36
Good	40.88	Over weight	13.39
Very good	50.96	Obese	2.58
**Characteristics of students**		**Smoking (2012)**	
**Age (2010)**	20.45	Never	71.47
**Gender (male) (2010)**	39.48	Had quit	8.60
**Migration background (2010)**		Sometimes	10.94
None	82.85	Daily	8.99
First generation	2.66	**Drinking (2012)**	
Second generation	7.01	(almost) never	13.56
Third+ generation	7.48	Once a month	17.99
**University of applied sciences (2010)**	22.42	2–3 per month	30.16
**Subject (2010)**		Once a week	25.75
Linguistics, cultural studies	28.54	2+ a week	12.55
Law	3.44	**Psychosocial factors**	
Economics	13.68	**Success (2012)**	
Social sciences	7.72	50/50 or lower	6.61
Mathematics, sciences	22.45	Somewhat likely	46.50
Medicine	4.45	Highly likely	46.89
Veterinary medicine^a^	2.43	**Support (2011)**	
Engineering	14.76	Not true at all–partly true	18.43
Arts and fine arts	2.54	Mostly true	51.65
**Parental background**		Exactly true	29.92
**ISEI (2010)**	56.29	**Self-esteem (2012)**	42.16
**Education (2010)**		**Material resources**	
Primary	9.37	**Manage cost of studies**	
Lower-secondary	27.70	Very/rather difficult	16.73
Upper-secondary	17.52	Neither nor	32.78
Tertiary education	45.41	Rather easy	37.46
		Very easy	13.03

Source: NEPS; n (2010): 15'244; n (2011): 10'787; n (2012): 11'920; ^a^including Agricultural-, forest- and nutrition sciences.

### 3.1. Health and health-related behavior

Fifty-one percent claimed to feel very good and 4 1% felt good, and 1% of students reported to feel bad or very bad. The health behavior of students reflected this good subjective health. Most of the students were of normal weight (78%), some were overweight (13%), less were underweight (6%) and only a few were obese (3%). The drinking behavior was moderate with over half of the students reporting to drink two or three times per month or less and only 10% reporting to drink two times or more per week. Ninety percent of students reported that they never smoked or that they had quit smoking, while 10% reported that they smoked sometimes or daily.

### 3.2. Psychosocial factors and material resources

Most students in our sample reported having good psychosocial resources. The likelihood to successfully complete the studies was rated as high or somewhat high by 47% respectively (93% combined), only 7% gave themselves only a 50/50 or lower chance. The social support among students was also rated rather high with 82% of students approving of the statement that students support each other. Also, the reported self-esteem among students was rather high. Most students reported that they had no problems to manage the cost of their studies.

### 3.3. Direct and indirect correlations between socio-economic status and self-rated health

Table 3 displays the results of the multilevel regression model on self-rated health. As they grew older, students rated their health less positively. Male students consistently reported a better health than female students. In the basic model, second generation migrants also reported less good self-rated health. Students of Universities of Applied Sciences also had worse health than students of Universities. There are some differences with regard to the correlation between study subjects and health. Students of Engineering, Economics, and Medicine reported better health over time compared to students of Linguistics and Cultural Studies. Once psychosocial resources and material conditions were controlled in the model, students of Social Sciences and Agricultural-, Forest- and Nutrition Sciences and Veterinary Medicine also displayed better health. Our main socio-economic variables were education and occupational status of parents. Education had no significant impact on health. But, a higher occupational status of parents improved the health status of their offspring consistently over nearly all different models. The introduction of financial resources diminished the impact of ISEI on health. The third model introduced variables measuring health-related behavior to our model. Smoking was negatively associated with health even in the younger age group of students. This is consistent for occasional and regular smokers. Drinking was only harmful for those students who drank almost never or only once a month. Students who drank once a week or more reported better health. Underweight and obese students had worse health compared to students who had normal weight.

**Table 3:** Multilevel linear regression of students' characteristics and their socio-economic background on their self-reported health status.

	**Basic**	**+SES**	**+Health**	**+Psycho**	**+Material**
**Characteristics of students**					
Age	−0.02^**^	−0.02^**^	−0.01^**^	−0.02^**^	−0.01^**^
	(0.00)	(0.00)	(0.00)	(0.00)	(0.00)
Gender (male)	0.09^**^	0.09^**^	0.09^**^	0.06^**^	0.05^**^
	(0.01)	(0.01)	(0.01)	(0.01)	(0.01)
**Migration background**					
None	Ref	Ref	Ref	Ref	Ref
First generation	0.01	0.02	0.04	0.10^*^	0.11^**^
	(0.04)	(0.04)	(0.04)	(0.04)	(0.04)
Second generation	−0.05^*^	−0.03	−0.01	0.01	0.01
	(0.03)	(0.03)	(0.03)	(0.02)	(0.02)
Third+ generation	0.01	0.01	0.01	0.02	0.02
	(0.02)	(0.02)	(0.02)	(0.02)	(0.02)
University of applied science	−0.07^**^	−0.06^**^	−0.03^+^	−0.04^*^	−0.04^**^
	(0.02)	(0.02)	(0.02)	(0.02)	(0.02)
**Subject**					
Linguistics, cultural studies	Ref	Ref	Ref	Ref	Ref
Law	0.04	0.03	0.03	0.04	0.03
	(0.03)	(0.03)	(0.03)	(0.03)	(0.03)
Economics	0.10^**^	0.10^**^	0.08^**^	0.08^**^	0.07^**^
	(0.02)	(0.02)	(0.02)	(0.02)	(0.02)
Social sciences	0.02	0.02	0.04	0.05^*^	0.05^*^
	(0.02)	(0.02)	(0.02)	(0.02)	(0.02)
Mathematics, sciences	0.00	0.00	−0.00	0.01	0.01
	(0.02)	(0.02)	(0.02)	(0.02)	(0.02)
Medicine	0.16^**^	0.15^**^	0.13^**^	0.13^**^	0.13^**^
	(0.03)	(0.03)	(0.03)	(0.02)	(0.02)
Veterinary medicine^a^	0.07	0.07	0.06	0.10^*^	0.10^*^
	(0.04)	(0.04)	(0.04)	(0.04)	(0.04)
Engineering	0.08^**^	0.08^**^	0.07^**^	0.09^**^	0.09^**^
	(0.02)	(0.02)	(0.02)	(0.02)	(0.02)
Arts and fine arts	0.03	0.02	0.03	0.04	0.05
	(0.04)	(0.04)	(0.04)	(0.04)	(0.04)
**Parental background**					
ISEI (/10)		0.16^**^	0.12^**^	0.10^*^	0.07^+^
		(0.05)	(0.05)	(0.04)	(0.04)
**Education**					
Primary		Ref	Ref	Ref	Ref
Lower-secondary		0.02	0.02	0.02	0.01
		(0.02)	(0.02)	(0.02)	(0.02)
Upper-secondary		0.02	0.02	0.01	0.00
		(0.02)	(0.02)	(0.02)	(0.02)
Tertiary education		0.01	0.01	−0.01	−0.02
		(0.02)	(0.02)	(0.02)	(0.02)
**Health behavior**					
**Smoking**					
Never			Ref	Ref	Ref
Stopped			−0.04^*^	−0.03	−0.02
			(0.02)	(0.02)	(0.02)
Sometimes			−0.08^**^	−0.06^**^	−0.06^**^
			(0.02)	(0.02)	(0.02)
Daily			−0.21^**^	−0.18^**^	−0.17^**^
			(0.02)	(0.02)	(0.02)
**Drinking**					
(almost) never			−0.07^**^	−0.06^**^	−0.06^**^
			(0.02)	(0.02)	(0.02)
Once a month			−0.04^**^	−0.03^*^	−0.03^*^
			(0.01)	(0.01)	(0.01)
2–3 per month			Ref	Ref	Ref
Once a week			0.02	0.02	0.01
			(0.01)	(0.01)	(0.01)
2+ a week			0.03^+^	0.02	0.02
			(0.0)	(0.02)	(0.02)
**BMI**					
Underweight			−0.11^**^	−0.10^**^	−0.10^**^
			(0.02)	(0.02)	(0.02)
Normal weight			Ref	Ref	Ref
Over weight			−0.12^**^	−0.11^**^	−0.11^**^
			(0.02)	(0.02)	(0.02)
Obese			−0.42^**^	−0.39^**^	−0.38^**^
			(0.03)	(0.03)	(0.03)
**Psychosocial factors**					
**Success**					
50/50 or lower				Ref	Ref
Somewhat likely				0.03^*^	0.03^*^
				(0.02)	(0.02)
Highly likely				0.06^**^	0.06^**^
				(0.02)	(0.02)
**Support**					
Not true at all–partly true				Ref	Ref
Mostly true				0.06^**^	0.06^**^
				(0.01)	(0.01)
				(0.02)	(0.02)
Self-esteem				0.03^**^	0.03^**^
				(0.00)	(0.00)
**Material resources**					
**Manage cost of studies**					
Very/rather difficult					−0.08^**^
					(0.01)
Neither nor					Ref
Rather easy					0.04^**^
					(0.01)
Very easy					0.06^**^
					(0.02)
**Model**					
Variance Level 1	0.22	0.22	0.20	0.18	0.18
	(0.00)	(0.00)	(0.00)	(0.00)	(0.00)
Variance Level 2	0.24	0.24	0.24	0.24	0.24
	(0.00)	(0.00)	(0.00)	(0.00)	(0.00)

Source: NEPS; n (Level 1): 24,394, n (Level 2) 8,898; ^+^p < 0.1; ^*^p < 0.05; ^**^p < 0.01; standard error in parenthesis; ^a^including Agricultural-, forest- and nutrition sciences.

Having more psychosocial resources was positively correlated with self-rated health. Students who rated their probability to succeed as somewhat or highly likely did also rate their health better. Students who perceived the likelihood to receive support from fellow students as more likely and those reporting higher self-worth also reported better health. Finally, financial resources were included in the model. It is clear that those students who reported no serious difficulties in financing their studies also reported better health, while those who found financing their studies very or rather difficult reported worse health over time.

### 3.4. The correlation between socio-economic status of parents and health-related behavior, psychosocial factors and material resources

In a next step, we analyzed the pathways between the socio-economic background of students and their health already described above. Tables 4–6 show the results of multinomial logistic regression models between the ISEI and education of parents and health-related behavior, psychosocial factors and material resources of students. We found that health-related behavior correlated with socio-economic status. Children whose parents had a higher occupational status or had completed tertiary education were more likely to be underweight, but less likely to be overweight or obese. These children were also less likely to smoke regularly. However, the drinking behavior did not follow these patterns. On the contrary, a higher socio-economic background correlated with a more regular drinking behavior. In addition, psychosocial resources also varied by socio-economic background. Students with a higher socio-economic background had a more favorable outlook at their likelihood of succeeding in their studies. They also had a more positive view of the social support they were receiving of fellow students and had a higher self-esteem. In addition, students from a higher socio-economic background reported less problems financing their studies.

**Table 4:** Multinomial logistic regression analyses between health-related behavior and socio-economic background of parents.

	**BMI**	**Underweight**	**Normal weight**	**Overweight**	**Obese**
ISEI	1.01^*^	Ref	0.99^*^	0.98^*^	
	(0.00)		(0.00)	(0.00)
**Education (ref** = **secondary)**					
Primary	1.19	Ref	0.98	1.31	
	(0.39)		(0.21)	(0.49)	
Tertiary	1.14	Ref	0.74^*^	0.66^*^	
	(0.11)		(0.04)	(0.08)	
**Alcohol**	**Almost never**	**Once a month**	**2–3 per month**	**Once a week**	**2**+ **a week**
ISEI	0.99^*^	0.99^*^	Ref	1.005^*^	1.01^*^
	(0.00)	(0.00)		(0.00)	(0.00)
**Education (ref** = **medium)**					
Low	4.13^*^	1.83^*^	Ref	0.72	1.43
	(0.86)	(0.42)		(0.20)	(0.44)
High	0.82^*^	0.88^*^	Ref	1.13^*^	1.55^*^
	(0.05)	(0.05)		(0.06)	(0.11)
**Smoking**	**Never**	**Stopped**	**Sometimes**	**Daily**	
ISEI	Ref	1.00	1.00	0.99^*^	
		(0.00)	(0.00)	(0.00)
**Education (ref** = **medium)**					
Low	Ref	1.07	0.73	0.88	
		(0.27)	(0.20)	(0.22)
High	Ref	0.93	0.97	0.81^*^	
		(0.07)	(0.06)	(0.06)

n (BMI-ISEI): 11'171; n (BMI-education): 11'956; n (Alcohol-ISEI): 11'296; n (Alcohol-education): 12'096; n (Smoking-ISEI): 11'302; n (Smoking-education): 12'104; ^*^p < 0.05; standard error in parenthesis; ISEI and Education estimated in separate models.

**Table 5:** Multinomial logistic regression analyses between psychosocial resources and socio-economic background of parents.

**Likelihood to succeed**	**50/50 or lower**	**Somewhat likely**	**Highly likely**
ISEI	Ref	1.01^*^	1.02^*^
		(0.00)	(0.00)
**Education (ref** = **medium)**			
Low	Ref	0.59^*^	0.39^*^
		(0.10)	(0.09)
Medium	Ref	Ref	Ref
High	Ref	1.34^*^	1.77^*^
		(0.07)	(0.12)
**Support**	**Not true at all–partly true**	**Mostly true**	**Exactly** **true**
ISEI	Ref	1.00	1.004^*^
		(0.00)	(0.00)
**Education (ref** = **medium)**			
Low	Ref	0.49^*^	0.54^*^
		(0.09)	(0.06)
High	Ref	1.00	1.02
		(0.11)	(0.07)
**Self-esteem**	**Low**	**Medium**	**High**
ISEI	Ref	1.00	1.01^*^
		(0.00)	(0.00)
**Education (ref** = **medium)**			
Low	Ref	0.70^+^	0.88
		(0.13)	(0.15)
High	Ref	1.00	1.15^*^
		(0.05)	(0.06)

n (Succeed-ISEI): 15'330; n (Succeed-education): 16'413; n (Support-ISEI): 10'076; n (Support-education): 10'762; n (Self-esteem-ISEI): 11'257; n (Self-esteem-education): 12'050; ^*^p < 0.05; standard error in parenthesis; ISEI and Education estimated in separate models.

**Table 6:** Logistic regression analyses between material resources and socio-economic background of parents.

**Manage cost of studies**	**Very/rather difficult**	**Neither nor**	**Rather easy**	**Very easy**
ISEI	0.99^*^	Ref	1.02^*^	1.03^*^
	(0.00)		(0.00)	(0.00)
**Education (ref** = **medium)**				
Low	1.11	Ref	0.93	1.07
	(0.17)		(0.14)	(0.24)
High	0.68^*^	Ref	1.47^*^	2.00^*^
	(0.03)		(0.06)	(0.13)

n (ISEI): 15'329; n (Education): 16'411; ^*^p < 0.05; standard error in parenthesis; ISEI and education estimated in separate models.

These results allowed us to create new variables that took into account whether a certain behavior or characteristic was positively, negatively, or not associated by the socio-economic background of parents. These new variables were used in a next step in the following multivariate models to reflect the indirect effect of socio-economic status and health.

Table 7 shows these indirect effects of health behavior, psychosocial resources, and financial background. A positive correlation of those variables with the parents' SES also resulted in a positive impact on health over time. A negative correlation had a negative impact on health, with the exception of BMI, where negative and positive status were bad for health. Given that underweight was associated with a higher social status and overweight/obese was associated with a lower social status, this was not surprising.

**Table 7:** Multilevel linear regression models for indirect effects of parents' occupational status *via* health behavior and psychosocial resources on students' self-rated health (separate models).

**Health behavior**		**Psychosocial characteristics**	
**Smoking**		**Perceived likelihood of success**	
Neutral	Ref	High status	0.15^**^
Low status	−0.20^**^		(0.03)
	(0.02)	Neutral	Ref
N (Observations)	24,127	N (Observations)	24,381
N (Respondents)	8,759	N (Respondents)	8,893
Variance (Level 1)	0.22 (0.00)	Variance (Level 1)	0.22 (0.00)
Variance (Level 2)	0.24 (0.00)	Variance (Level 2)	0.24 (0.00)
**Drinking**		**Perceived likelihood of social support**	
High status	0.00	High status	0.07^**^
	(0.01)		(0.01)
Neutral	Ref	Neutral	Ref
Low status	−0.07^**^	N (Observations)	23,623
	(0.02)	N (Respondents)	8,621
N (Observations)	24,127	Variance (Level 1)	0.21 (0.00)
N (Respondents)	8,759	Variance (Level 2)	0.24 (0.00)
Variance (Level 1)	0.22 (0.00)	**Self-worth**	
Variance (Level 2)	0.24 (0.00)	High status	0.21^**^
**BMI**			(0.21)
High status	−0.09^**^	Neutral	Ref
	(0.03)	N (Observations)	24,117
Neutral	Ref	N (Respondents)	8,756
Low status	−0.21^**^	Variance (Level 1)	0.21 (0.00)
	(0.02)	Variance (Level 2)	0.24 (0.00)
N (Observations)	24,085	**Material resources**	
N (Respondents)	8,738	**Manage cost of studies**	
Variance (Level 1)	0.21 (0.00)	High status	0.09^**^
Variance (Level 2)	0.24 (0.00)		(0.01)
		Neutral	Ref
		Low status	−0.11^**^
			(0.02)
		N (Observations)	24,385
		N (Respondents)	8,893
		Variance (Level 1)	0.21 (0.00)
		Variance (Level 2)	0.24 (0.00)

Source: NEPS ^+^p < 0.1; ^*^p < 0.05; ^**^p < 0.01; controlled for age, gender, migration status, type of university and study-subject; standard error in parenthesis.

## 4. Discussion

This study explored the impact of the parental socio-economic status on the health status of university students based on cohort 5 of the National Educational Panel Study. Our study contributed to the literature in several ways. Surprisingly, little was known about the general health of university students and even less research on students' health was based on a large scale representative survey. Most studies either focused on specific health behavior of students (39), on specific groups, such as medical students (40), or on mental health only (8). We found that, overall, university students were in good health with over 90 % of the students reported to be of good and very good health in their first study year. Students in NEPS were therefore healthier than in other surveys and they also showed less symptoms of potentially harmful health-related behavior (4, 8).

Our study was especially important with regard to health inequality. Most students are in a privileged situation. They are in the process of acquiring an education that will most likely give them access to high-ranking jobs and a good income. Although the expansion of education in the last decades had improved the access to higher education across all social classes, there was still a bias toward children from a higher socio-economic background being more likely to be enrolled at universities. We found that the importance of the socio-economic status of parents for the health of their offspring was still persistent among university students, a connection that was already known for adolescents (41) and young children (42). This showed that the “social class” of origin had more persistence than the aspired social class as suggested by Hagquist (14). However, we found no impact of parental education on students' health. This was in line with other studies who also found that education of parents had no correlation with health, once education of children was accounted for (43). Yet, in our analyses, the occupational status of parents was related to children's health. An interesting observation in this context was that once material conditions of students were introduced to the model, the coefficient of ISEI was reduced. This was an important finding as it showed that material conditions might be a mediator between occupation of parents and health of students. These differences between measures of socio-economic position of families and the interfering role of the own SEP of the student are an important finding for future research on the influence of parental background on children's health.

As it is often found that inequality has a strong indirect effect on health (15, 16), one main aspect of our analysis was the analysis of indirect effects of family background *via* material resources, psychosocial factors, and health-related behavior on students' health. Our analysis reproduced previous findings and confirmed that the socio-economic conditions of parents had an indirect impact on health of students *via* behavioral, psychosocial, and material factors. Students' health behavior, their psychosocial and material resources showed clear patterns depending on their socio-economic background (described above). Education and the occupational status of parents mattered, more or less, equally for the indirect effect. This suggests that different possible mediating pathways should be taken into account while analyzing life-course related health inequalities, otherwise the importance of parental education would have not been noticed, due to a non-significant direct effect.

Our study had some shortcomings. NEPS only contained self-rated health as a health measure. Therefore, we were not able to extend our analysis with a measure of psychological health like depression, which is an important indicator for students' wellbeing (10). It would have been also important to control for measures of stress as an important co-founder for health (25). Some measures had also not been available across all waves and had to be forwarded between waves.

Another caveat was the missing of a direct measure of financial conditions of students or their parents. Especially a measure of students' income would have been an important information. As with all panel data, we might had issues with panel mortality and selection: It is likely that especially students who drop out are the ones less likely to continue with their studies. This potential selection of students who did better in their studies should have resulted in a more homogeneous group of students with less impact of their social background, since students with higher educated parents typically do better in university. That health inequality still exists speaks to the importance of inequality for health research.

One final limitation is our approach to mediation analysis. As was pointed out by Richiardi et al. (44) and VanderWeele (45), mediation analyses can be biased and other methods, such as counterfactual analysis would be a better alternative do disentangle mediation. Therefore, our specific mediation analysis only gave us only an impression of the indirect effect of parental socio-economic status on student health. Due to the fact that the mediator variables were categorical variables with multiple categories we also couldn't apply a standard mediation analysis and were therefore not able to calculate total effects and determine the magnitude and significance of the indirect effects.

The importance of the life course for health outcomes later on in life is widely known (46). Especially the theory of cumulative inequality and the critical period model for health inequality are important in this regard. The time between adolescence and adulthood marks an important period (1) and can be seen as a “critical period.” For many, this is the phase where they leave their parental homes and start their own life away from home. It is also the time before the so called “rush-hour” of life starts, where people have to start their career, find a partner and start a family, build a home, etc. (47). The health inequalities we found in university students are likely to accumulate over time and differences between social groups may even increase over time, resulting in greater inequality and worsened health outcomes later on in life.

We believe our study is an important contribution to the understudied subject of students' health. We see the impact of social inequality on health among such a privileged group like university students as an important sign of the importance of health inequality. Given that more and more students in Germany come from a non-academic background, health inequality is likely to become more important in the future. And health promotion of students should be of special interest for universities and for public health.

## Data availability statement

Publicly available datasets were analyzed in this study. This data can be found here: https://www.neps-data.de/.

## Ethics statement

Ethical review and approval was not required for the study on human participants in accordance with the local legislation and institutional requirements. Written informed consent to participate in this study was provided by the participants' legal guardian/next of kin.

## Author contributions

CD wrote the manuscript and conducted the data analysis. KD, JS, MR, WS, PR, ND, and CRP contributed in writing the manuscript and participated in the final review of the paper. All authors contributed to the article and approved the submitted version.
